# High Resistance of *Salmonella* spp. and *Shigella* spp. in Blood and Stool Cultures from the Sukraraj Tropical and Infectious Disease Hospital, Kathmandu, Nepal, 2015−2019

**DOI:** 10.3390/tropicalmed6020059

**Published:** 2021-04-23

**Authors:** Anup Bastola, Prajjwal Pyakurel, Rajan Bikram Rayamajhi, Saugat Shrestha, Pruthu Thekkur, Basudev Pandey, Parmananda Bhandari, Anu Maharjan, Jeffrey K. Edwards

**Affiliations:** 1 Sukraraj Tropical and Infectious Disease Hospital, Ministry of Health and Population, Government of Nepal, Teku, Kathmandu 44600, Nepal; drbasupandey@gmail.com (B.P.); parmananda_bhandari@yahoo.com (P.B.); 2 School of Public Health and Community Medicine, B.P. Koirala Institute of Health Sciences, Dharan 56700, Nepal; prajjwal.pyakurel@bpkihs.edu; 3 World Health Organization (WHO) Country Office, Lalitpur 44700, Nepal; rayamajhir@who.int (R.B.R.); shresthasau@who.int (S.S.); 4 Centre for Operational Research, International Union Against Tuberculosis and Lung Disease (The Union), 75006 Paris, France; pruthu.tk@theunion.org; 5 The Union South-East Asia Office, New Delhi 110016, India; 6 Central Department of Microbiology, Tribhuvan University, Kirtipur, Kathmandu 44618, Nepal; mhjanu14@gmail.com; 7 Department of Global Health, University of Washington, Seattle, WA 98195, USA; jeffrey.edwards@fmridaho.org

**Keywords:** *Salmonella* spp., *Shigella* spp., antimicrobial resistance, Nepal, operational research

## Abstract

Antimicrobial resistance (AMR) is an increasing global concern, particularly in Southeast Asian countries like Nepal. The aim of this study was to determine the proportion of *Salmonella* spp. and *Shigella* spp. among culture-positive bacterial isolates in blood and stool samples from 2015 to 2019 and their AMR pattern. Routinely collected data were abstracted from medical records and laboratory electronic databases of the Sukraraj Tropical and Infectious Disease Hospital (STIDH), Kathmandu, Nepal. All culture-positive bacterial isolates from blood and stool samples were included in the study. Among 390 blood cultures positive for bacterial isolates, *Salmonella* spp. were isolated in 44%, with *S.* Typhi being the most frequent (34%). Antibiotic resistance was demonstrated among *Salmonella* spp. to ciprofloxacin (68%), ofloxacin (16%), amoxicillin (13%) and cotrimoxazole (5%). Of the 357 stool cultures positive for bacterial isolates, the proportion of *Shigella* spp. isolated was 31%. Antibiotic resistance among *Shigella* spp. was demonstrated to cotrimoxazole (59%), tetracycline (40%), amoxicillin (38%) and ciprofloxacin (25%). *Salmonella* spp. and *Shigella* spp. were the most predominant organisms among all the bacterial isolates in blood and stool cultures, respectively. Nalidixic acid was the antibiotic to which both *Salmonella* spp. and *Shigella* spp. were most resistant.

## 1. Introduction

Antimicrobial resistance (AMR) is a rapidly emerging concern, affecting health systems and economies globally [1,2]. AMR complicates the treatment of infection and is associated with increased mortality and morbidity [3]. The Global Antimicrobial Surveillance System (GLASS) of the World Health Organization (WHO) tracks antimicrobial resistance occurrences in 22 nations with suspected bacterial infections, and *Salmonella spp*. have been among the most commonly reported resistant bacteria [4].

Southeast Asian countries are known to contribute to AMR secondary to multiple factors such as rapid intensification of food production systems, poorly controlled public access to antimicrobials, health practitioners, the animal health industry, irrational use of antimicrobials and the practice of self-medication. In recent years, these countries have undergone unprecedented economic development and are a potential hub contributing to the global spread of AMR as bacteria can easily be transmitted by movement of people from one region to another and through the international trade of animals and products to other parts of the globe [5].

Nepal is challenged by the progression of AMR within the health sector for multiple reasons, including (1) inappropriate prescribing by providers, (2) lack of access to providers and availability of antimicrobials over the counter, (3) lack of medication compliance, (4) drug company influencing provider prescribing and (5) inadequate infection prevention and control practices within healthcare facilities [6]. To address these issues, Nepal endorsed a National Antimicrobial Resistance Containment Action Plan in 2016 [7].

Factors driving AMR have resulted in Gram-negative enteric pathogens (especially *Salmonella* spp. and *Shigella* spp.) to emerge as highly resistant pathogens to multiple antimicrobials, including broad-spectrum quinolones, and are now under WHO-supervised surveillance within Nepal [7]. Both *Salmonella* spp. and *Shigella* spp. are important water-borne bacteria that are prevalent in Nepal, especially in urban settings such as Kathmandu [8]. These organisms are among the most common pathogens isolated in blood and stool samples, respectively, from patients presenting with fever and acute gastroenteritis in healthcare settings. 

For acute undifferentiated fever, most likely related to salmonellosis, the Nepali 2016 infectious disease guidelines recommend to include cefixime, ceftriaxone, azithromycin or amoxicillin. Similarly, antibiotic treatment guidance for diarrhoea or dysentery associated with shigellosis generally includes a combination of ceftriaxone, ciprofloxacin, ofloxacin or cotrimoxazole with metronidazole, tinidazole or secnidazole [9]. A study in Kathmandu by Joshi et al. (2018) demonstrated that *Salmonella* spp. showed high multidrug resistance to broad-spectrum quinolones among cultured isolates (ciprofloxacin 45% and levofloxacin 14%) [10]. Bhetwal et al. (2017) demonstrated *Salmonella* spp. resistance rates of 37% and 55% to ciprofloxacin and ofloxacin, respectively [11]. Similarly, a study done by Khan et al. (2013), looking at *Shigella* spp. isolates from infected children in Kathmandu Valley, demonstrated broad resistance to multiple drugs (nalidixic acid 96%, ampicillin 86%, cotrimoxazole 83% and ciprofloxacin 48%) [12]. A recent study done by Shakya et al. (2016) indicated multidrug resistance among 46% of patients with shigellosis in a Kathmandu hospital [13].

Nepal’s antimicrobial resistance surveillance systems are insufficient to capture the possible of antimicrobial resistance at the beginning and to the molecular level of the resistance mechanism [7]. The AMR country report of 2018 mentions the data collection of eight pathogens for GLASS and includes *Salmonella* spp. and *Shigella* spp. [14]. There remains limited information about how antimicrobial resistance patterns have changed over time for these two frequently infectious entero-pathogens in Nepal and represents a significant gap in AMR knowledge, which will likely have important treatment ramifications [7]. Hence, we proposed to evaluate the sensitivity pattern and trend of these entero-pathogens that would likely provide insights and guide healthcare providers, hospital leadership and policy makers in improving antimicrobial stewardship going forward in the country.

The aim of this study was to determine the proportion of *Salmonella* spp. and *Shigella* spp. among all culture-positive bacterial isolates from blood and stool samples from 2015 to 2019 and to determine their AMR patterns against different antibiotics. 

## 2. Materials and Methods

### 2.1. Study Design

This was a hospital-based, retrospective study done using secondary data.

### 2.2. General Setting

Nepal is a developing landlocked country in South Asia, with a population of approximately 30 million. It lies between China and India, in the centre of the Himalayas, and is characterised by a largely rural population. The primary urban centre is Kathmandu Valley, which has an estimated 1.5 million inhabitants. Nepal is classified as a low-income country and has a struggling public healthcare system [15]. The Nepalese government is implementing a new public healthcare insurance strategy to improve access to care and treatment. The program is currently implemented in 46 districts, and 7 districts are scheduled for expansion [16]. However, success remains limited, and the Nepali population continues to obtain the majority of healthcare from private practitioners and institutions [17,18].

### 2.3. Specific Setting

The study was carried out at the Sukraraj Tropical and Infectious Disease Hospital (STIDH), Teku, Kathmandu, Nepal. The STIDH was founded in 1933 and is a national referral hospital for Nepal, specialising in infectious and tropical diseases [19]. It has 100 beds and provides outpatient and inpatient services, including isolation wards, Intensive Care Unit (ICU) care and laboratory services. Laboratory services include microbiology, haematology, parasitology, immunology, biochemistry, CD4 count and real-time polymerase chain reaction (PCR). 

External quality control is completed twice within a year by the National Public Health Laboratory (NPHL), the central laboratory in the country. Samples provided by the NPHL are processed in the hospital’s lab and are reported to the NPHL. Laboratory services are provided 7 days a week, 24 hours a day. The STIDH manages all major infectious diseases, including typhoid fever (salmonellosis) and shigellosis. Sterility testing of culture media is done by incubating a prepared medium of each batch, and performance testing of media and antibiotics is carried out using the standard control strain of *Escherichia coli* (ATCC 25922).

### 2.4. Collection and Processing of Samples and Antibiotic Susceptibility Testing (AST) of the Isolates

#### 2.4.1. Blood Sample

Blood samples of 10 mL were inoculated into 50 ml of Brain Heart Infusion (BHI) broth. The broth was then incubated at 37 °C for 24−48 h, then sub-cultured on blood agar (BA) and MacConkey Agar (MA) and incubated at 37°C for 18−24 h. The BA plates were incubated in a microaerophilic atmosphere (5−10% CO_2_) using a candle jar. After incubation, the MA plates were observed for non-lactose fermenting colonies and BA plates for non-haemolytic colonies. Presumptive identification was done by colony characteristics, Gram staining and biochemical reactions. For serotype identification, serotyping was done by the agglutination method [20]. 

AST was done by modified Kirby–Bauer disc diffusion method, as recommended by the Clinical Laboratory Standard Institute (CLSI). The antibiotic discs used were ampicillin (10 µg), amoxicillin (30 µg), cotrimoxazole (25 µg), nalidixic acid (30 µg), cefixime (5 µg), cefotaxime (30 µg) ceftriaxone (30 µg), chloramphenicol (30 µg), ciprofloxacin (5 µg) and ofloxacin (5 µg) [21].

#### 2.4.2. Stool Sample

Stool (2 g) was collected in sterile containers. All specimens were promptly transported to the laboratory within 2 h of collection. The samples were inoculated for culture onto MA and xylose lysine deoxycholate (XLD) agar. The samples were then incubated at 37 °C for 18−24 h. The identification of *Salmonella* spp. was done by colony morphology, Gram staining, biochemical tests and serotyping. Similarly, the identification of *Shigella* spp. was based on colony morphology, Gram staining and biochemical reactions [20].

Antimicrobial susceptibility was evaluated by modified Kirby–Bauer disc diffusion method following CLSI guidelines. AMR was evaluated by using antibiotic discs of ampicillin (10 µg), amoxicillin (30 µg), cotrimoxazole (25 µg), nalidixic acid (30 µg), ciprofloxacin (5 µg), ofloxacin (5 µg), levofloxacin (5 µg), cefixime (5 µg), cefotaxime (30 µg) and ceftriaxone (30 µg) [21].

We extracted the antibiotic sensitivity pattern of the isolates through the laboratory database and divided them into two categories, sensitive and resistant. All the isolates that showed intermediate susceptibility were included as resistant in this study.

### 2.5. Study Population and Period

The study population for *Salmonella* spp. included all the culture-positive *Salmonella* spp. among 390 blood cultures positive for all bacterial isolates from 2015 to 2019. Similarly, the study population for *Shigella* spp. included all the culture-positive *Shigella* spp. among 357 stool cultures positive for all bacterial isolates from 2015 to 2019.

### 2.6. Study Variables

The study variables used in the study were age, gender and year of sample collection.

### 2.7. Data Collection, Analysis and Statistics

Data were extracted from record books (2015−2016) and the laboratory electronic database (2017−2019) using a standardised performa and maintained in Microsoft Excel 2016 (Redmond, WA, USA). Then, the data were anonymised, and a unique identifier (patient’s identification number) was used and double-entry validation was completed.

The yearly trend in culture positivity for bacterial isolates from blood samples and the proportion of *Salmonella* spp., along with the trend in culture positivity for bacterial isolates from stool samples and the proportion of *Shigella* spp. from 2015 to 2019, were determined.

### 2.8. Research Ethics

Ethical approval for the study was obtained from the Ethics Advisory Group of the International Union against Tuberculosis and Lung Disease, Paris, France (EAG number: 57/19) and from the Nepal Health Research Council Ethical Review Board (Ref no. 1502). Permission to conduct the study at the STIDH was obtained from the hospital’s director. 

## 3. Results

Out of 750, 1873, 2843, 2113 and 3777 total blood samples submitted annually, the proportion with bacterial growth (culture positivity) was 0.7%, 0.9%, 1.6%, 9.0% and 3.5% in the years 2015, 2016, 2017, 2018 and 2019, respectively. 

There were 390 blood samples with culture growth for different bacterial isolates (Table 1). Among them, *S.* Typhi accounted for the majority of infections (33.8%), followed by *S.* Paratyphi (9.7%). The proportion of other blood isolates is mentioned in Supplementary Table S1. In total, the proportion of culture growth for *Salmonella* spp. was 44.1%. The proportion of *Salmonella* spp. was the highest in the age group of <21 years (71.2%), followed by 21−40 years (42.3%). Males showed the predominant proportion of culture positivity for *Salmonella* spp. (46.9%). Culture positivity for *Salmonella* spp. was significantly higher in the year 2016 (100%) than in other years, which varied from 31% to 56%. However, there were relatively few samples obtained from 2015 to 2016 (22 samples were collected in total).

Out of 530, 1453, 1805, 1350 and 641 stool samples submitted annually, the proportion of culture positivity was 2.1%, 13.8%, 3.1%, 4.9% and 3.6% in the years 2015, 2016, 2017, 2018 and 2019, respectively. Among them, the proportion of *Shigella* spp. and *Salmonella* spp. among bacterial isolates from 2015 to 2019 is shown in Table 2. There were 357 stool samples positive for different bacterial isolates. The proportion of other stool isolates is mentioned in Supplementary Table S1. Among them, the proportion of *Salmonella* spp. accounted for the majority of isolates (44.5%), followed by *Shigella* spp. (31.4%). *Salmonella* spp. were more frequent in the age group of 41-60 years (56.5%), followed by 21-40 years (52.2%). *Shigella* spp. isolates were seen more frequently in the age group of <21 years (49.3%). However, the proportion of *S.* Typhi (3.9%) and *S*. Paratyphi (3.9%) was negligible in the stool samples. Culture positivity for *Salmonella* spp. was higher among females (45.5%). There was a significantly higher proportion of culture-positive samples in the year 2016 (56.3%) versus other years (3.1−18.5%). In addition, there was a higher proportion of samples with *Salmonella* spp. in 2016 (54.2%) versus other years (26–35%).

Other species included *Aeromonas* spp., *Enterobacter cloacae*, *E. faecalis*, *Klebsiella pneumoniae*, *Proteus* spp., *Providencia* spp., *Pseudomonas* spp., *Vibrio cholerae* O1, *V. fluvialis*, *V. parahaemolyticus*, *Vibrio spp.* and *Vibrio* other than O1 and O139.

Table 3 shows the antimicrobial resistance pattern of *Salmonella* spp. and *Shigella* spp. isolated from blood and stool samples from January 2015 to December 2019. From blood culture isolates, 61.4% *S.* Typhi and 68.41% *S.* Paratyphi were resistant to ciprofloxacin. Similarly, 71.2% *S*. Typhi and 68.4% *S.* Paratyphi was resistant to nalidixic acid. Similarly, nalidixic acid (58.9%) and cotrimoxazole (58%) showed the highest resistance pattern among *Shigella* spp. isolated from the stool samples.

**Figure 1:**
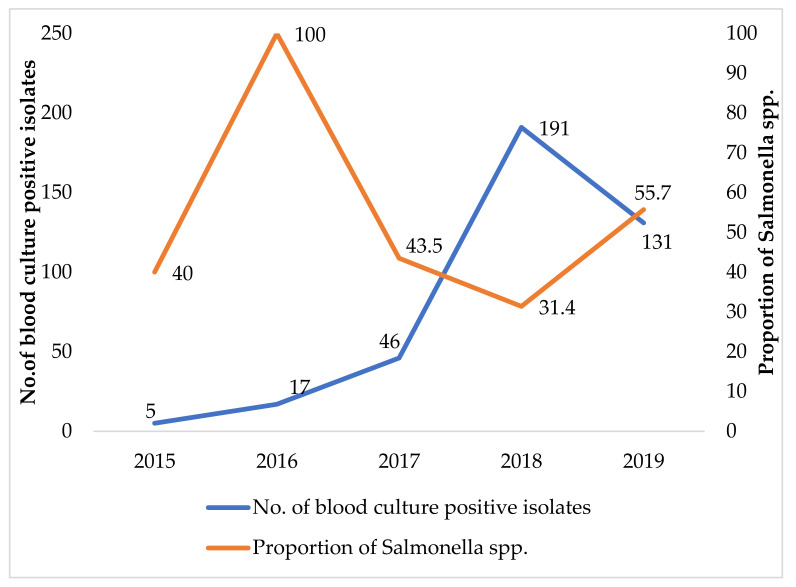
Trend of blood culture-positive isolates and proportion of *Salmonella* spp. at the STIDH, Kathmandu, Nepal, from 2015 to 2019.

Figure 1 shows the trend in blood samples with culture-positive bacterial isolates in culture and the proportion of *Salmonella* spp. at the STIDH from 2015 to 2019. The blood samples’ positivity illustrates a gradual increase over the period of three years (2015 to 2017). In 2016, a total of 17 bacterial isolates were 100% positive for *Salmonella* spp. The culture positivity in the year 2018 showed a sharp rise, with a total of 191 samples, of which 31.4% were positive for *Salmonella* spp.

**Figure 2:**
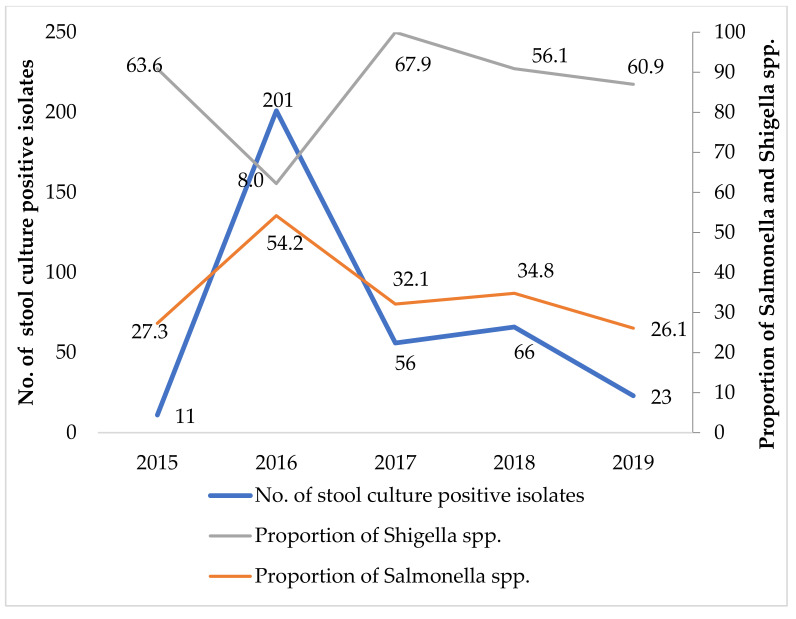
Trend of stool culture-positive isolates and proportion of *Shigella* spp. and *Salmonella* spp. at the STIDH, Kathmandu, Nepal, from 2015 to 2019.

Figure 2 shows the trend in the number of stool culture bacterial isolates and the proportion of *Salmonella* spp. and *Shigella* spp. isolated from 2015 to 2019 at the STIDH. The proportion of culture positivity for *Salmonella* spp. was the highest in the year 2016 (54.2%), followed by 2018 (34.8%). Similarly, the proportion of culture positivity for *Shigella* spp. was the highest in the year 2017 (67.9%), followed by 2015 (63.6%). Except for 2016, the proportion of *Shigella* spp. isolated was greater than that of *Salmonella* spp. in stool samples among bacteria isolated from 2015 to 2019.

## 4. Discussion

This study assessed the AMR pattern and trends in the leading tropical and infectious disease hospital in Nepal. The key findings of the study were as follows: (1) There was a higher proportion of *Salmonella* spp. in blood cultures among all the bacterial isolates from 2015 to 2019 (44.1%); (2) there was a greater proportion of *Shigella* spp. and *Salmonella* spp. in stool cultures among all the bacterial isolates (31.4% and 44.5%, respectively); (3) *Salmonella* spp. in blood cultures and *Shigella* spp. in stool cultures were found the most resistant to nalidixic acid; (4) the proportion of *Salmonella* spp. isolated from blood samples was the highest in the year 2016 (100%), followed by the year 2019 (55.7%); and (5) the proportion of *Shigella* spp. and *Salmonella* spp. isolated from stool samples was the highest in 2017 (67.9%) and 2016 (54.2%), respectively.

This study is important as the majority of cases of salmonellosis and shigellosis are caused by contaminated food and water, which is crucial in the context of Kathmandu V, where there is a lack of health literacy, frequent poor hand hygiene and inadequate waste management and sewage systems.

Our study adds additional insights to the existing evidence of commonly isolated pathogens (*Salmonella* spp. and *Shigella* spp.) and the challenging scenario of irrational antibiotic use within Nepal. These results hopefully will encourage the national AMR Committee, the Ministry of Health and Population (MoHP) and the Government of Nepal (GoN) to focus upon developing improved guidance on the rational use of antibiotics and antibiotic stewardship.

In our study, out of 11,356 blood samples submitted, 390 (3.4%) were positive for both Gram-positive and Gram-negative bacterial isolates. As the STIDH is a national referral hospital for infectious diseases, patients with a wide spectrum of infectious diseases, including HIV, malaria, brucellosis, dengue, scrub typhus and tuberculosis, visit the hospital, and blood samples are taken for surveillance culture in all febrile illnesses. This might be the possible reason for the low proportion of culture positivity seen in blood cultures. Other reasons might include a history of prior antibiotic use before sample submission and inconsistency in the sample volume taken by the laboratory staffers.

A higher proportion of *S.* Typhi were seen among blood culture isolates in our study, which is similar to a study done by Bhetwal et al. [11] and Joshi et al. [10]. A likely cause for this high proportion of *Salmonella* infections is directly related to contaminated drinking water sources [22].

The highest proportion of *Salmonella* spp. among blood culture-positive isolates was found in the age group of <21 years in our study, which differs from the study done by Joshi et al. in 2018 [10], where the highest proportion of *Salmonella* spp. was seen in the age group of 21−40 years. These differences among the age groups could be due to the sample population and geographical distribution of cases but may also suggest that other issues are in play. Our study showed the highest proportion of *Salmonella* spp. among males. This finding is consistent with the studies done by Joshi et al. and Umair and Siddiqui and likely reflects increased risk for this population, perhaps related to lapses in hygiene, particularly in lower age groups [10,23].

Out of 5779 stool samples submitted, 357 (6.2%) were positive for both Gram-positive and Gram-negative bacteria. We found that the proportion of *Shigella* spp. and *Salmonella* spp. in stool cultures was 31.4% and 44.5%, respectively, which is comparatively higher than an earlier study done in Kathmandu by Kansakar et al. However, the two studies differ in the time period and length of analysis of five versus three years, respectively. *Shigella* spp. isolates were seen more frequently in the adolescent age group in our study. A study conducted by Liu et al. in China, showed a higher proportion of shigellosis in the adult population [24]. Unhygienic practices among children make them vulnerable to food- and waterborne diseases.

In our study, *Salmonella* spp. isolated from blood were found most resistant to nalidixic acid (*S*. Typhi 71% and *S*. Paratyphi 68%), followed by ciprofloxacin (*S.* Typhi 61% and *S.* Paratyphi 68%) and ofloxacin (*S.* Typhi 0.8% and *S.* Paratyphi 15.8%). These findings are different compared with the study completed by Shrestha et al., where 83% and 3.6% of *Salmonella* spp. were resistant to nalidixic acid and ciprofloxacin, respectively, whereas none were resistant to ofloxacin [25]. Similarly, 58.9%, 25% and 17.9% of *Shigella* spp. isolated from stool were found most resistant to nalidixic acid, ciprofloxacin and ofloxacin, respectively. This finding is slightly different from the findings of Khan et al. Likewise, *Salmonella* spp. from stool samples was found most resistant to amoxicillin (14.5%), followed by nalidixic acid (13.2%), tetracycline (9.4%) and ciprofloxacin (6.9%), which is different from the study done by Kansakar et al. Nalidixic acid resistance is considered a marker of increased minimum inhibitory concentration (MIC) for fluoroquinolones and also portrays reduced susceptibility of fluoroquinolones against *Salmonella* spp. [26]. Irrational use of antibiotics contributes significantly to the increasing rate of drug resistance in developing countries. Additionally, the development of reduced susceptibility or resistance to fluoroquinolones may be due to mutation in genes encoding DNA gyrase (*gyrA* and *gyrB*) and topoisomerase IV (*parC* and *parE*) and plasmid-mediated quinolone resistance [27].

The proportion of *Salmonella* spp. isolated from blood was the highest in the year 2016. Likewise, there was a significantly higher number of stool samples submitted by symptomatic patients in 2016 as well. The likely explanation for these variations is the 7.8 magnitude earthquake in 2015, which severely affected Kathmandu Valley and led to catastrophic disruption of many basic services across the region [28].

### Strengths and Limitations

This study included all blood and stool samples submitted to the STIDH laboratory from 2015 to 2019. Data validation was completed through a review of all related medical records and a standardised laboratory electronic database. Lastly, this study followed the international Strengthening the Reporting of Observational studies in Epidemiology (STROBE) guidelines for observational studies [29]. 

The primary limitation of this study is an apparent lack of consistent reporting of blood and stool culture samples during the time period, as reflected in the large variation in the number of samples recorded each year. This may have led to significant sampling bias and impacted our results. The possible reasons for the sample size variability are a combination of a fragile reporting and record system, a lack of motivated staff to document thoroughly and leadership challenges at multiple levels. This, in turn, needs to be improved through an improved reporting and recording system and a mechanism to promote evidence-based research, in addition to incentivising health professionals and building a strong AMR surveillance system. 

Further research needs to be completed to confirm our results. In addition, laboratory system process analysis could help ease some of the reporting challenges and lead to an improved, more robust reporting system as both are necessary to enhance AMR surveillance and stewardship within Nepal and decrease the risk of worsening health outcomes for the population in Nepal and elsewhere.

The findings of this study can be used to improve clinical guidelines by preparing policy briefs and reporting to the AMR committee, the MoHP and the Government of Nepal. Additionally, the findings could also be used as a catalyst to develop a more robust, standardised AMR surveillance and reporting system across multiple healthcare facilities in Nepal.

## 5. Conclusions

*Salmonella* spp. and *Shigella* spp. were the most commonly isolated pathogens among all the bacterial isolates from blood and stool cultures from 2015 to 2019 at the STIDH, Kathmandu, Nepal. These isolates were mostly resistant to quinolones and fluoroquinolones. On the contrary, there was a low proportion of *Salmonella* spp. resistant to classical drugs like ampicillin/amoxicillin, cotrimoxazole and chloramphenicol. Similarly, the proportion of *Shigella* spp. resistant to ampicillin, cotrimoxazole, chloramphenicol and tetracycline was also low. Drug-resistant *Salmonella* spp. and *Shigella* spp. represent an increasing challenge for Nepal. Enhancement should be done on laboratory diagnosis and data recording, evidence-based guideline development in antibiotic prescription, national AMR surveillance, AMR stewardship, research on AMR and evidence-based policy formulation to combat AMR. 

## Figures and Tables

**Table 1:** Proportion of *Salmonella* spp. among all bacterial isolates from blood cultures at STIDH, Kathmandu, Nepal, January 2015 to December 2019.

Characteristics	No. of Culture-Positive Isolates		*S.* Typhi		*S.* Paratyphi		*Salmonella* spp.^±^	
	*N*	(%) *	*N*	(%) ^#^	*N*	(%) ^#^	*N*	(%) ^#^
Total	390	(100)	132	(33.8)	38	(9.7)	172	(44.1)
Age in years								
<21	111	(28.5)	59	(53.2)	20	(18.0)	79	(71.2)
21−40	175	(44.9)	59	(33.7)	14	(8.0)	74	(42.3)
41−60	74	(19.0)	9	(12.2)	4	(5.4)	14	(18.9)
>60	26	(6.7)	2	(7.7)	0	(0.0)	2	(7.7)
Missing	4	(1.0)	3	(75.0)	0	(0.0)	3	(75.0)
**Gender**								
Male	239	(61.3)	84	(35.1)	27	(11.3)	112	(46.9)
Female	151	(38.7)	48	(31.8)	11	(7.3)	60	(39.7)
**Year of sample submission**								
2015	5	(1.3)	1	(20.0)	1	(20.0)	2	(40.0)
2016	17	(4.4)	12	(70.6)	5	(29.4)	17	(100)
2017	46	(11.8)	17	(37.0)	3	(6.5)	20	(43.5)
2018	191	(49.0)	48	(25.1)	11	(5.8)	60	(31.4)
2019	131	(33.6)	54	(41.2)	18	(13.7)	73	(55.7)

CDST, culture and drug susceptibility testing. *: Column percentage. **^±^** two isolates were *S*. typhimurium; ^#^ row percentage.

**Table 2:** Proportion of *Shigella*, *Salmonella* and other species among bacterial isolates from stool culture at the STIDH, Kathmandu, Nepal, from January 2015 to December 2019.

Characteristics	No. of Culture-Positive Isolates		*Shigella* spp.^±^		*Salmonella* Typhi ^±^		*Salmonella* Paratyphi ^±^		*Salmonella* spp.^±^		Other Species	
	N	(%) *	N	(%) ^#^	N	(%) ^#^	N	(%) ^#^	N	(%) ^#^	N	(%) ^#^
Total	357	(100)	112	(31.4)	14	(3.9)	14	(3.9)	159	(44.5)	88	(24.6)
Age in years												
<21	71	(19.9)	35	(49.3)	1	(1.4)	0	(0.0)	14	(19.7)	22	(31.0)
21−40	136	(38.1)	31	(22.8)	5	(3.7)	6	(4.4)	71	(52.2)	36	(26.5)
41−60	85	(23.8)	22	(25.9)	6	(7.1)	4	(4.7)	48	(56.5)	15	(17.6)
>60	55	(15.4)	20	(36.4)	2	(3.6)	4	(7.3)	23	(41.8)	12	(21.8)
Missing	10	(2.8)	4	(40.0)	0	(0.0)	0	(0.0)	3	(30.0)	3	(30.0)
**Gender**												
Male	155	(43.4)	51	(32.9)	4	(2.6)	7	(4.5)	67	(43.2)	38	(24.5)
Female	202	(56.6)	60	(30.2)	10	(5.0)	7	(3.5)	92	(45.5)	50	(24.8)
**Year of sample submission**												
2015	11	(3.1)	7	(63.6)	0	(0.0)	0	(0.0)	3	(27.3)	1	(9.1)
2016	201	(56.3)	16	(8.0)	12	(6.0)	6	(3.0)	109	(54.2)	77	(38.3)
2017	56	(15.7)	38	(67.9)	1	(1.8)	5	(8.9)	18	(32.1)	0	(0.0)
2018	66	(18.5)	37	(56.1)	1	(1.5)	2	(3.0)	23	(34.8)	7	(10.6)
2019	23	(6.4)	14	(60.9)	0	(0.0)	1	(4.3)	6	(26.1)	3	(13.0)

CDST-culture and drug susceptibility testing. *: Column percentage; ^#^ row percentage; ^±^ 2 samples were positive for both *Salmonella* spp. and *Shigella* spp.

**Table 3:** Antimicrobial resistance pattern of *Salmonella* spp. and *Shigella* spp. among culture-positive isolates at the STIDH, Kathmandu, Nepal, from 2015 to 2019.

Drugs	*Salmonella* Typhi (Blood Sample)		*Salmonella* Paratyphi (Blood Sample)		*Shigella* spp. (Stool Sample)		*Salmonella* spp. (Stool Sample)	
	N	(%) *	N	(%) *	N	(%) *	N	(%) *
Total	132	(100)	38	(100)	112	(100)	159	(100)
Ampicillin	4	(3.0)	1	(2.6)	5	(4.5)	4	(2.5)
Cotrimoxazole	4	(3.0)	2	(5.3)	65	58	6	(3.8)
Nalidixic acid	94	(71.2)	26	(68.4)	66	(58.9)	21	(13.2)
Ceftriaxone	0	(0.0)	0	(0.0)	8	(7.1)	0	(0.0)
Cefotaxime	0	(0.0)	0	(0.0)	0	(0.0)	0	(0.0)
Chloramphenicol	6	(4.5)	0	(0.0)	12	(10.7)	0	(0.0)
Ciprofloxacin	81	(61.4)	26	(68.4)	28	(25.0)	11	(6.9)
Ofloxacin	1	(0.8)	6	(15.8)	20	(17.9)	7	(4.4)
Levofloxacin	0	(0.0)	0	(0.0)	0	(0.0)	0	(0.0)
Tetracycline	0	(0.0)	0	(0.0)	45	(40.2)	15	(9.4)
Amoxicillin	11	(8.3)	5	(13.2)	42	(37.5)	23	(14.5)
Cefixime	1	(0.8)	0	(0.0)	4	(3.6)	0	(0.0)
Azithromycin	7	(5.3)	1	(2.6)	0	(0.0)	0	(0.0)
Gentamicin	0	(0.0)	0	(0.0)	0	(0.0)	1	(0.6)
Amikacin	0	(0.0)	0	(0.0)	0	(0.0)	0	(0.0)

* Column percentage for each of the antibiotics. Note: Two *S*. typhimurium species identified in blood samples are not included in the table.

## Data Availability

Data can be made available on request to corresponding author.

## Supplementary Materials

The following are available online at https://www.mdpi.com/article/10.3390/tropicalmed6020059/s1, Table S1: List of isolates from blood and stool samples, Table S2: MDR *Salmonella* spp. from blood, Table S3: MDR *Shigella* spp. from stool.
