# Supplementary material for: High Resistance of Salmonella spp. and Shigella spp. in Blood and Stool Cultures from the Sukraraj Tropical and Infectious Disease Hospital, Kathmandu, Nepal, 2015−2019
Source: Trop Med Infect Dis. 2021 Apr 23;6(2):59. doi: 10.3390/tropicalmed6020059 (PMC8167633; doi:10.3390/tropicalmed6020059)
Supplement: Supplementary file 1 [file tropicalmed-06-00059-s001.zip › tropicalmed-1150350-supplementary.pdf]

Tropical Medicine and Infectious Disease

MDPI

Supplementary

Table S1. List of isolates from blood and stool samples.

Isolates from blood	n	%	Isolates from stool	n	%
S. Typhi	132	33.8	*S.* Typhi	14	3.9
S. Paratyphi	38	9.7	*S.* Paratyphi	14	3.9
S. Typhimurium	2	0.5	*Salmonella* (Other than *S.* Typhiand *S.* Paratyphi)	131	36.5
S. aureus	118	30.3	*Shigella* spp.	112	31.2
A. baumannii	7	1.8	*Proteus* spp.	12	3.3
Pseudomonas spp.	4	1.0	*Aeromonas* spp.	1	0.3
Citrobacter spp.	12	3.1	*Providencia* spp.	1	0.3
K. pneumoniae	6	1.5	*E. cloacae*	3	0.8
E. coli	20	5.1	*V. cholerae O1*	42	11.7
CONS	32	8.2	*Vibrio other than O1 and O139*	10	2.8
Alpha hemolytic streptococci	1	0.3	*Vibrio spp.*	2	0.5
S. pneumoniae	1	0.3	*V. parahaemolyticus*	12	3.3
E. aerogenes	1	0.3	*V. fluvialis*	1	0.3
S. sonnei	2	0.5	*Pseudomonas* spp.	2	0.6
Contaminated	13	3.3	*K. pneumoniae*	1	0.3
Mixed growth	1	0.3	*E. faecalis*	1	0.3
**Total**	**390**	**100**	**Total**	**359**	**100**

Table S2. MDR *Salmonella* spp. from blood.

Organisms	MDR n (%)	Non-MDR n (%)
S. Typhi	4 (3.0)	128 (97.0)
S. Paratyphi	0 (0.0)	38 (100)
S. Typhimurium	0 (0.0)	2 (100)

Table S3. MDR *Shigella* spp. from stool.

Antibiotics	No. of *Shigella* spp.
AMX+NA+TET	4
AMX+NA+CFM	1
AMX+COT+TET	4
C+COT+TET	3
COT+TET+CTR	3
COT+NA+TET	8
NA+TET+GEN	1
AMX+COT+NA+TET	10
AMX+COT+NA+CFM	1
C+COT+NA+TET	1
AMX+COT+NA+CIP+OF	5
AMX+NA+CIP+OF+TET	1
C+COT+NA+CIP+OF	1
AMX+C+COT+NA+CIP+OF	2
AMX+C+COT+NA+CIP+OF+TET	3
**Total**	**48**

Note: AMX- Amoxicillin; AMP- ampicillin; C- Chloramphenicol; COT- Cotrimoxazole; NA- Nalidixic acid; CIP- Ciprofloxacin; OF- Ofloxacin; CFM- Cefixime; CTR- Ceftriaxone; GEN- Gentamicin; TET- Tetracycline
